# Supplementary figures and images for: Crystal structure and nanobodies against domain 3 of the malaria parasite fusogen Plasmodium falciparum HAP2
Source: Biochem J. 2026 Jan 22;483(2):119–33. doi: 10.1042/BCJ20250297 (PMC12905498; doi:10.1042/BCJ20250297)

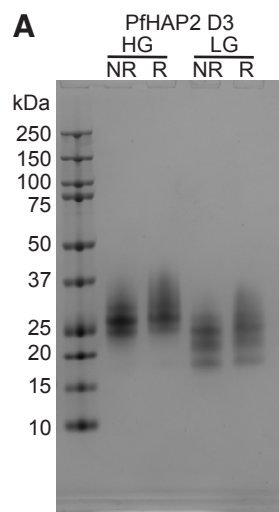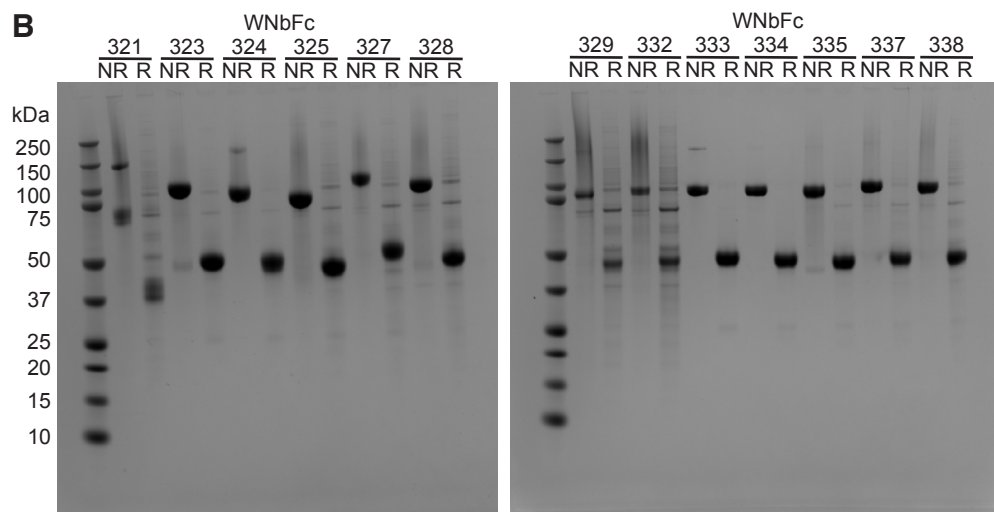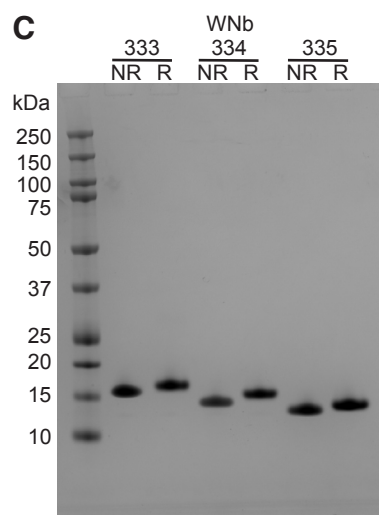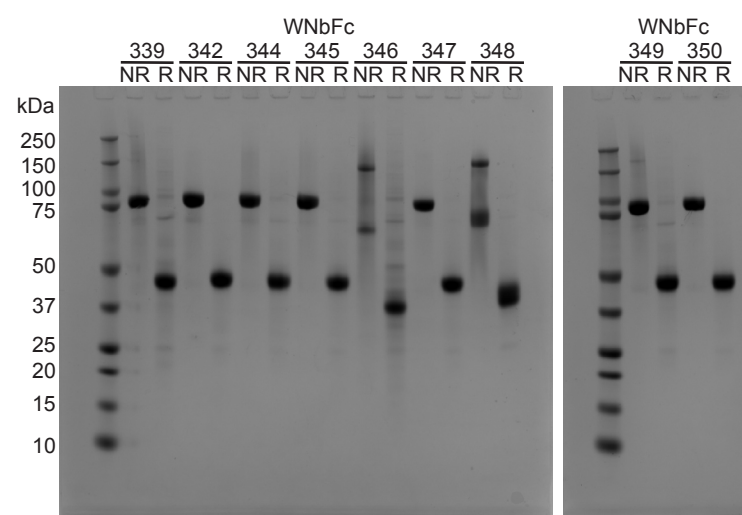

Supplement: online supplementary figure 1 [file bcj-483-2-BCJ20250297-s001.pdf]

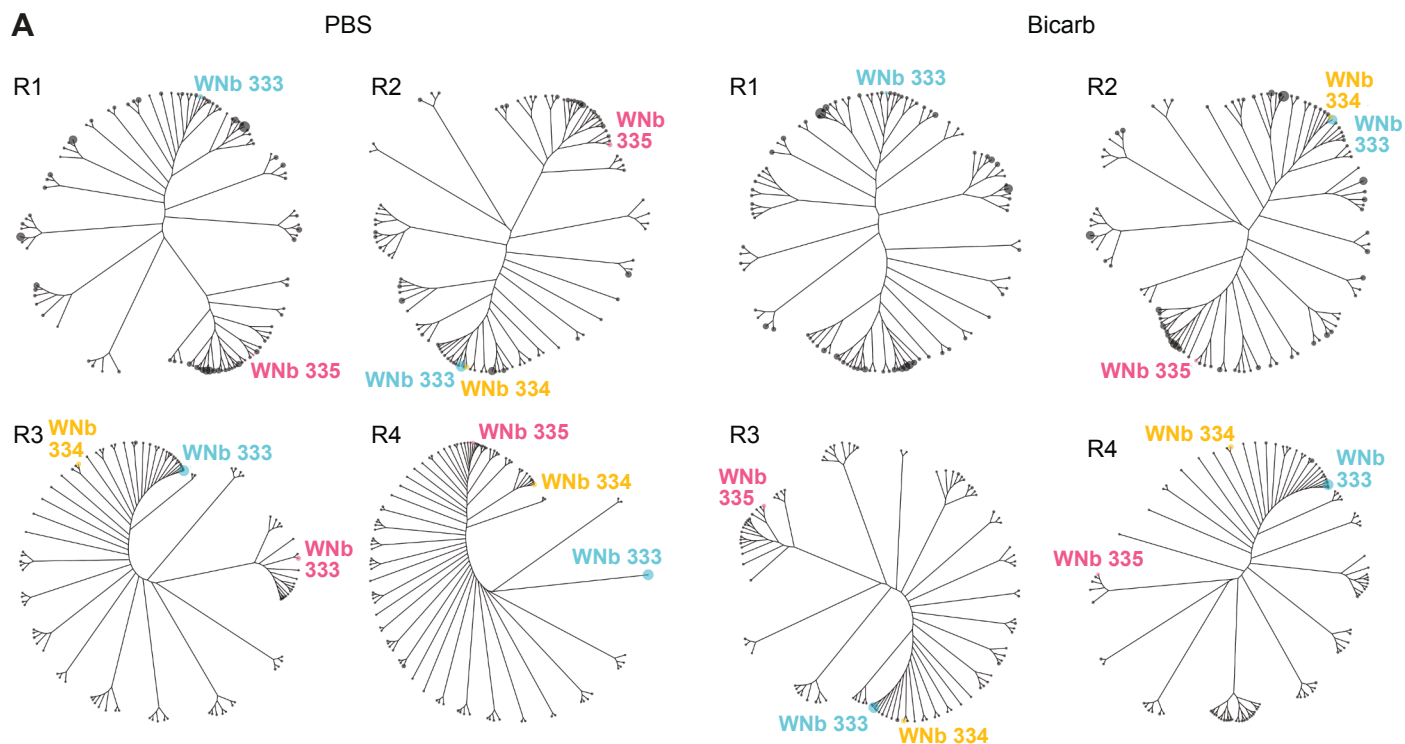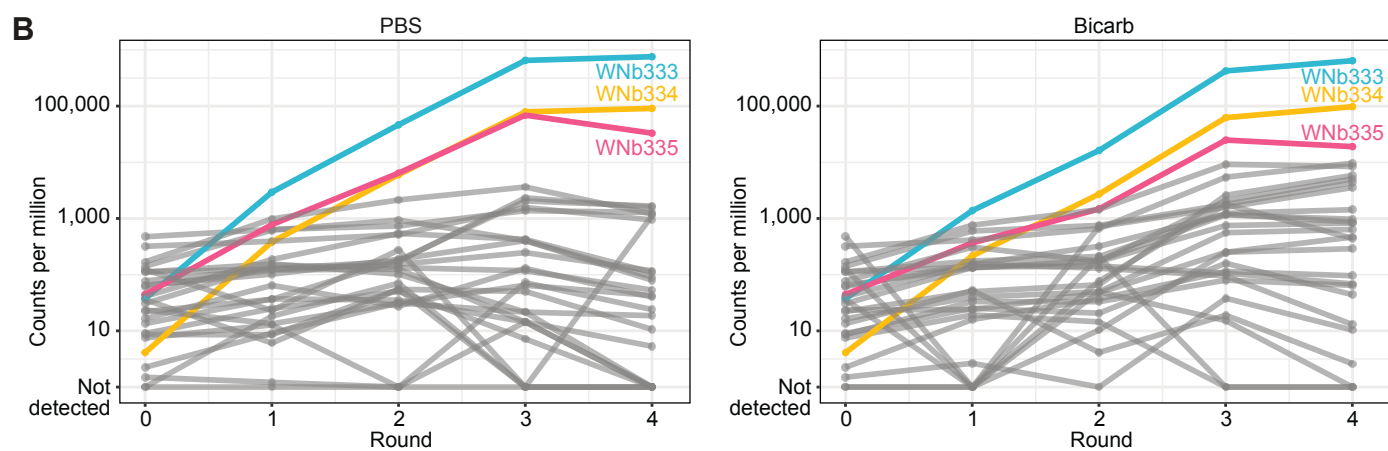

Supplement: online supplementary figure 2 [file bcj-483-2-BCJ20250297-s002.pdf]

**A**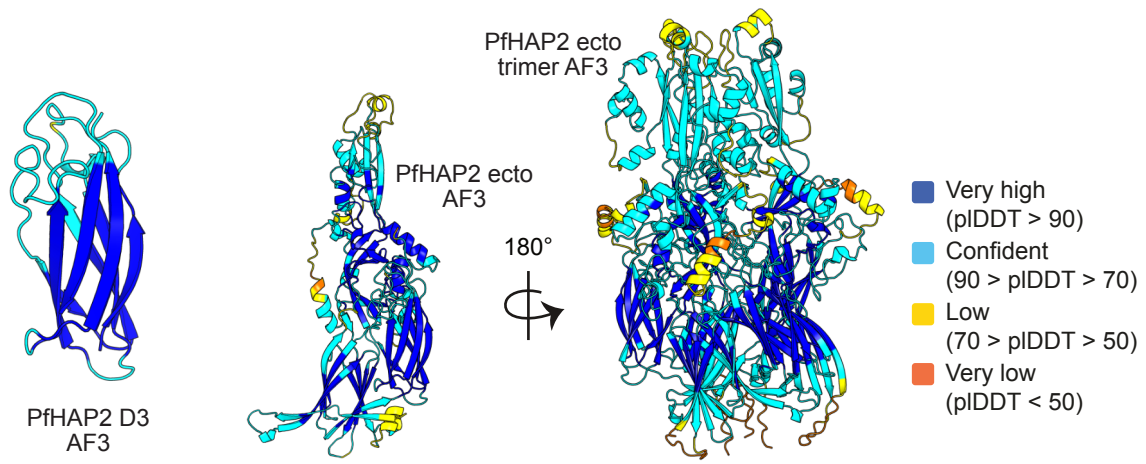**B**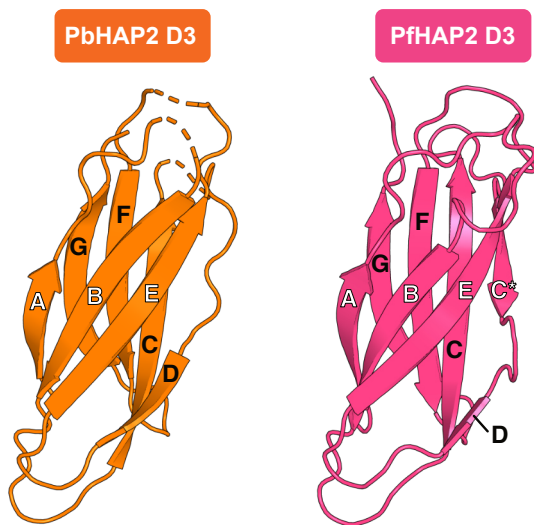**C**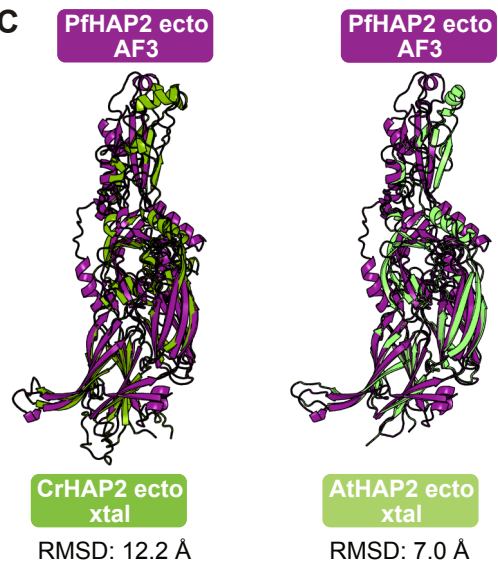

Supplement: online supplementary figure 4 [file bcj-483-2-BCJ20250297-s004.pdf]
