# Supplementary material for: Crystal structure and nanobodies against domain 3 of the malaria parasite fusogen Plasmodium falciparum HAP2
Source: Biochem J. 2026 Jan 22;483(2):119–33. doi: 10.1042/BCJ20250297 (PMC12905498; doi:10.1042/BCJ20250297)
Supplement: online supplementary table 1 [file bcj-483-2-BCJ20250297-s005.docx]

Table S1. Amino acid sequences of nanobodies (Nb) isolated against PfHAP2 D3.

| **Nb** | **Full Nb sequence** |
| --- | --- |
| WNb321 | QVQLQMNSLKLEDTAVYYCNRVLSLVREWGQGTQVTVSS |
| WNb322 | QVQLQESGGGLVQPGGSLRLSCAVSGFSFSNYWMNWVRQAPGKGLEWVAGFKSTTGSTYQGETMKGRFTISRDNAKNTLDLQMNSLKPEDTAVYYCARIIGGSVWSAMDYWGKGTQVTVSS |
| WNb323 | QVQLQESGGGLVQPGASLNLSCAASPFVLNYYRIGWFRQAPGKQRDLVATISSGGSTNYADSVKGRFTISRDSAKNTLYLEMSSLKPEDTAVYYCNVGSMWFGKILNYWGQGTQVTVSS |
| WNb324 | QVQLQESGGGLVQPGGSLRLSCAASGRIRSSYVMSWYRQGPGKERELVDPVTDRFTISRDDAKSTVFLQIDSLKPEDTALYSCARIPRNSNWRTRDNFDARGQGTQVTVSS |
| WNb325 | QVQLQESGGGLVQPGGSLRLSCAASGRLFRLYAMGWYRQAPGKQRELVAVIAGGGTINYADSVKGRFTISRDNTKNTVYLQMNSLKPEDTAVYYCNARWAWYDYWGQGTQVTVSS |
| WNb326 | QVQLQESGGGLVQPGGSLRLSCSASGFTFSRYWMDWVRQAPGKGLEWVSVINSENGGTYYADSVKGRFTMSRDDAKNMLYLQMNSLAPEDTAVHYCTKHEWGSRWSIGSWGQGTQVTVSS |
| WNb327 | QVQLQESGGGWVQPGGSLRLSCVASGSIFSIYYMNWYRQSPGKQRELVASISRNGTTNYADSVKGRFSIWREKAKNTVYLEMNSLKPEDTAVYYCVKDVSWTTMVPILLGSWGQGTQVTVSS |
| WNb328 | QVQLQESGGGLVRAGESLRLSCAASGGTFSLHTMRWFRQALGKEREFVAGISRSGGRTSYADTAKGRFTISRDNAKNTVYLQMNSLKPEDTAVYSCKASRAVPTIRGYEYTYWGQGTQVTVSS |
| WNb329 | QVQLQESGGGLVQPGGSLRLSCATSGLTFSAYWMHWVRQPPGKGLEWVSALNKYGNTYYSDSVKGRFTISRDGAKNTLYLEMNYLESDDTAVYYCVAPGTSGWALMNYWGKGTQVTVSS |
| WNb330 | QVQLQESGGGLVQPGGSLRLSCVASGFTFGSSYMTWVRQAPGKGPEWVSSIHADLRTFYTNSVQGRFAISRDVAENTLYLQMNSLKPEDTALYYCARDGINWFTRDYWGQGTQVTVSS |
| WNb331 | QVQLQESGGGLVQPGGSLRLSCAASGFTFSSYWMNWVRQALGKGLEWVSTIDNSGTHSGYADSVKGRFTVARDNAKNTVYLQMTSLKPDDTAVYYCTNWEGLAGLGMDYWGKGTQVTVSS |
| WNb332 | QVQLQESGGGLVQPGGSLRLSCAASGFTFSSRYMTWYRRAPGKERELVAVIGIWGATNYADSVKGRFAISRDNAKNTVYLQMNSLKPEDTAVYFCNAVHLWARQGGYWGQGTQVTVSS |
| WNb333 | QVQLQESGGGSVQPGGSLRLSCAASGFTLEDYAIGWFRQAPGKGREGISCISSTDGSTHYADSAKGRFTISRDDAKNTAYLQMNSLKVEDTDVYYCAALSQEHMITVQRMCAVPAAHYWGRGTQVTVSS |
| WNb334 | QVQLQESGGGLVQPGGSLRLSCAASGFTLDDYAIGWFRQDPGKGREGVSCISLSDGSTYYADAVTGRFTISRDNAKDTVYLQMNSLKPEDTAVYYCAALSQKQMTTVQAMCAVPIANYWGQGTQVTVSS |
| WNb335 | QVQLQESGGGLVQPGGSLRLSCEASASGTIVALGTMAWYRQAPGNQRELVATITEESSTYYADSVKGRCTISRDNARNTMYLRLNSLKPEDTAVYYCNLANFWGQGTQVTVSS |
| WNb336 | QVQLQESGGGLVQPGGSLILSCVASGFTFSRYAYGWARQVAGKGLEWVSGIFGDGKTYYSDSVKGRFTIPRDNAKNTAYLRMNSLNSEDTAVYYCAALGHTRWEYDYWGQGTQVTVSS |
| WNb337 | QVQLQESGGGLVQAGGSLRLSCAASGRSFSTYNTGWFRQAPGKERRFVAAITWSGADIYYADSVKGRFTISRDNAKNTVYLQMNSLKPEDTAVYYCAKFNADEYDYWGQGTQVTVSS |
| WNb338 | QVQLQESGGGLVQPGGSLRLSCVYPTTIFSIFAMGWYRQAPGKQREWVAGITRSGITNYADSVKGRFTISRDNAKNTLYLQMNSLKPEDTAVYYCARGSSGFWSLRGQGTQVTVSS |
| WNb339 | QVQLQESGGGLVQAGGSLRLSCAASGRTFSNYAMGWFRQAPGKEREFVAAISWSGGSTYYADSVKGRFTISRDNAKNTAYLQMNSLKPEDTAVYYCRSFRSWGQGTQVTVSS |
| WNb340 | QVQLQESGGGLVQPGGSLTLSCAASAFTFSRYWMNWVRQAPGKGLEWVSAITSGGSSTYYADSVKGRFDISRDNAKNTLYLQMNSLKPEDTAVYYCAKSWGGLYNGMDYWGKGTQVTVSS |
| WNb341 | QVQLQESGGGLVQPGGSLRLSCAASGFTFSRYAMAWVRQAPGKGLEWVSDIFNSGGSTYYADSVKGRFTISGDNAKNTLYLQMNSLKPEDTAVYYCAKDRYGSSWLNYGMDYWGKGTQVTVSS |
| WNb342 | QVQLQESGGGLVQAGGSLRLSCAASGRTLSTLRTEPRMGWFRQAPGKGPEWVSTISTGGRRLYYADSVKGRFTISRDNAKNTVYLQMNSLKPEDTAMYFCVARAYGTSYRGKGTQVTVSS |
| WNb343 | QVQLQESGGGLVHAGGSLRLSCAASGSIFSRSAMDWYRQAPGKQRELVAHITTVDSTKYADSVKGRFTISRDNAENTVYLQMSSLKPEDTAVYYCNDDPSGFMAMTRRPSDYWGQGTQVTVSS |
| WNb344 | QVQLQESGGGLVQPGGSLRLSCAASGGFFRFYTMGWYRQAPGKQRQLVATITPTGFTNYADAVKGRFTISRDNAKNTVYLQMNSLKPEDTAVYYCNSGWWLSQPDFGSWGQGTQVTVSS |
| WNb345 | QVQLQESGGGLVQPGGSLRLSCAASGFTFSNYAMSWVRQAPGKGPEWVSTISPGGRRLYYADSVKGRFAISRDNAKNTLYLQMNSLKPEDTAVYYCNARYLSVGWNYWGQGTQVTVSS |
| WNb346 | QVQLQMDNLKPEDTAVYYCNANRVIVIAGAWNYWGQGTQVTVSS |
| WNb347 | QVQLQESGGGLVQFGGSLRLSCAASGRRFSNYGLNWYRQAPGKQRELVATITSGGSTDYADSVKGRFTISRDNAKNTLYLQMNSLKPEDTGVYYCARWVWVGRWGQGTQVTVSS |
| WNb348 | QVQLQMNSLKPEDTALYSCARIPRNSNWRTRDNFDARGQGTQVTVSS |
| WNb349 | QVQLQESGGGLVQPGGSLRLSCTASGFTFSRRVMSWYRQAPGKEREFVAFITTTGDSTNYADFAKGRFTIARDNAKNTVYLQMNDLKSEDTAVYYCVQGGAWHSGSYYYVWPFASWGQGAQVTVSS |
| WNb350 | QVQLQESGGGLVQPGGSLRLSCTASRNIFSISAMGWYRQAPGKERELVASINKWSGITNYADSVKGRFTISRDNAKNTVYLQMNSLKPEDTAMYYCNARRFGDWYDTWGQGTQVTVSS |
