# Supplementary material for: Crystal structure and nanobodies against domain 3 of the malaria parasite fusogen Plasmodium falciparum HAP2
Source: Biochem J. 2026 Jan 22;483(2):119–33. doi: 10.1042/BCJ20250297 (PMC12905498; doi:10.1042/BCJ20250297)
Supplement: online supplementary table 2 [file bcj-483-2-BCJ20250297-s006.docx]

Table S2. Affinities of nanobody-Fcs for PfHAP2 D3 by bio-layer interferometry (BLI).

|  | ***K*_D_ (nM)** | ***k*_a_ (x10^5^ M^-1^s^-1^)** | ***k*_d_ (x10^-5^ s^-1^)** | **Full *X*^2^** | **Full *R*^2^** |
| --- | --- | --- | --- | --- | --- |
| WNb333 | 15.8 ± 1.3 | 3.6 ± 0.5 | 567.8 ± 35.7 | 0.008 ± 0.003 | 0.996 ± 0.001 |
| WNb334 | 26.1 ± 0.1 | 4.4 ± 0.4 | 1144.5 ± 105.5 | 0.013 ± 0.001 | 0.995 ± 0.001 |
| WNb335 | 50.8 ± 0.9 | 1.9 ± 0.2 | 990.6 ± 121.4 | 0.006 ± 0.001 | 0.996 ± 0.001 |

Mean affinities (*K*_D_), association rates (*k*_a_), dissociation rates (*k*_d_), *X*^2^ and *R*^2^ values of two independent experiments are given with standard deviation.
