# Supplementary material for: Crystal structure and nanobodies against domain 3 of the malaria parasite fusogen Plasmodium falciparum HAP2
Source: Biochem J. 2026 Jan 22;483(2):119–33. doi: 10.1042/BCJ20250297 (PMC12905498; doi:10.1042/BCJ20250297)
Supplement: online supplementary table 3 [file bcj-483-2-BCJ20250297-s007.docx]

Table S3. Summary of interactions between PfHAP2 D3 and WNb 334 using PISA.

| PfHAP2 D3 | Group | WNb 334 | Group | Distance (Å) |
| --- | --- | --- | --- | --- |
|  |  |  |  |  |
| *Hydrogen bonds* | | | | |
| Thr 507 | O | Arg 45 | N | 3.1 |
| Ile 509 | N | Pro 114 | O | 3.1 |
| Ile 509 | O | Ala 116 | N | 3.4 |
| Ile 511 | N | Ala 116 | O | 3.0 |
| Ile 511 | O | Asn 117 | ND2 | 3.0 |
| Pro 512 | O | Gln 103 | NE2 | 3.0 |
| Pro 512 | O | Asn 117 | ND2 | 3.4 |
| Asn 534 | ND2 | Gly 42 | O | 3.7 |
| Lys 616 | N | Lys 102 | O | 3.5 |
| Other WNb 334 interfacing residues (PfHAP2 D3) | | | | |
| Ser 502 | Tyr 503 | Thr 505 | Ile 506 | His 508 |
| Thr 510 | Lys 513 | Cys 515 | His 530 | Trp 532 |
| Leu 609 | Ser 610 | Phe 611 | Asn 612 | Leu 613 |
| Thr 614 | Ser 615 |  |  |  |
| Other PfHAP2 D3 interfacing residues (WNb 334) | | | | |
| Tyr 32 | Gln 39 | Pro 41 | Lys 43 | Gly 44 |
| Leu 99 | Ser 100 | Thr 105 | Val 113 | Ile 115 |
| Tyr 118 | Trp 119 |  |  |  |
